# Urinary metabolite profiling and risk of progression of diabetic nephropathy in 2670 individuals with type 1 diabetes

**DOI:** 10.1007/s00125-021-05584-3

**Published:** 2021-10-22

**Authors:** Stefan Mutter, Erkka Valo, Viljami Aittomäki, Kristian Nybo, Lassi Raivonen, Lena M. Thorn, Carol Forsblom, Niina Sandholm, Peter Würtz, Per-Henrik Groop

**Affiliations:** 1 Folkhälsan Institute of Genetics, Folkhälsan Research Center, Helsinki, Finland; 2 Department of Nephrology, University of Helsinki and Helsinki University Hospital, Helsinki, Finland; 3 Research Program for Clinical and Molecular Metabolism, Faculty of Medicine, University of Helsinki, Helsinki, Finland; 4 Nightingale Health Ltd, Helsinki, Finland; 5 Department of General Practice and Primary Health Care, University of Helsinki and Helsinki University Hospital, Helsinki, Finland; 6 Department of Diabetes, Central Clinical School, Monash University, Melbourne, VIC Australia

**Keywords:** Diabetic nephropathy, Metabolite profiling, NMR, Progression, Type 1 diabetes

## Abstract

**Aims/hypothesis:**

This prospective, observational study examines associations between 51 urinary metabolites and risk of progression of diabetic nephropathy in individuals with type 1 diabetes by employing an automated NMR metabolomics technique suitable for large-scale urine sample collections.

**Methods:**

We collected 24-h urine samples for 2670 individuals with type 1 diabetes from the Finnish Diabetic Nephropathy study and measured metabolite concentrations by NMR. Individuals were followed up for 9.0 ± 5.0 years until their first sign of progression of diabetic nephropathy, end-stage kidney disease or study end. Cox regressions were performed on the entire study population (overall progression), on 1999 individuals with normoalbuminuria and 347 individuals with macroalbuminuria at baseline.

**Results:**

Seven urinary metabolites were associated with overall progression after adjustment for baseline albuminuria and chronic kidney disease stage (*p* < 8 × 10^−4^): leucine (HR 1.47 [95% CI 1.30, 1.66] per 1-SD creatinine-scaled metabolite concentration), valine (1.38 [1.22, 1.56]), isoleucine (1.33 [1.18, 1.50]), pseudouridine (1.25 [1.11, 1.42]), threonine (1.27 [1.11, 1.46]) and citrate (0.84 [0.75, 0.93]). 2-Hydroxyisobutyrate was associated with overall progression (1.30 [1.16, 1.45]) and also progression from normoalbuminuria (1.56 [1.25, 1.95]). Six amino acids and pyroglutamate were associated with progression from macroalbuminuria.

**Conclusions/interpretation:**

Branched-chain amino acids and other urinary metabolites were associated with the progression of diabetic nephropathy on top of baseline albuminuria and chronic kidney disease. We found differences in associations for overall progression and progression from normo- and macroalbuminuria. These novel discoveries illustrate the utility of analysing urinary metabolites in entire population cohorts.

**Graphical abstract:**

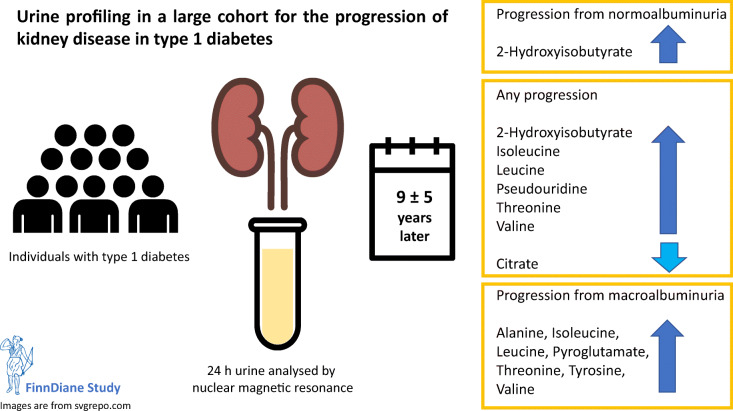

**Supplementary Information:**

The online version contains supplementary material available at 10.1007/s00125-021-05584-3.

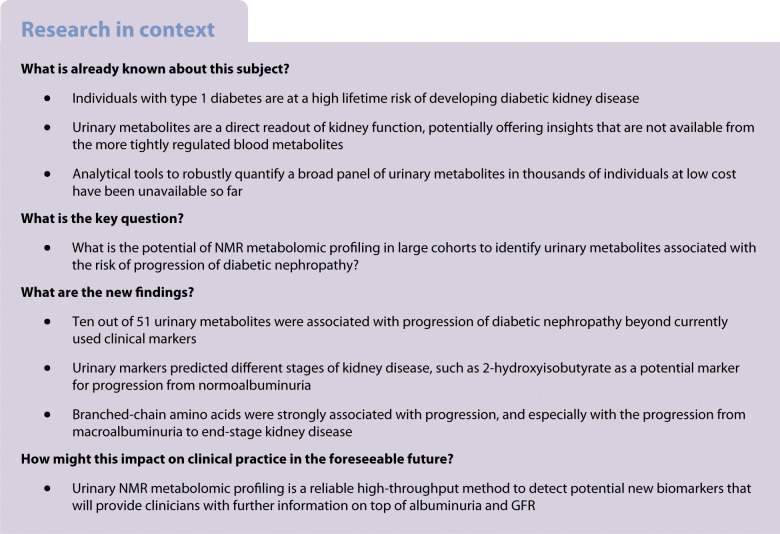



## Introduction

Type 1 diabetes usually manifests at a young age. Therefore, the affected individuals are at an especially high lifetime risk of developing diabetic complications such as diabetic nephropathy, which affects a third of individuals and is the main cause of higher mortality rates in type 1 diabetes [1]. Although the molecular understanding of diabetic nephropathy is expanding, its exact pathophysiology is still elusive [2]. Urinary metabolites are a direct readout of kidney function [3] and might therefore offer novel molecular insights into the pathophysiology of diabetic nephropathy.

Detailed metabolic profiling, also known as metabolomics, has been successfully used to identify blood metabolites that are involved at different stages of diabetic nephropathy [4]. Metabolites in blood are strongly regulated, whereas the metabolite concentrations in urine are more variable and, therefore, may offer insights that are not attainable from the blood [5]. Metabolomic profiling of the urine led, for example, to the discovery of the role of formate in BP regulation, a role that had not been discovered in blood [6]. The metabolites found in the urine relate to pathways from cardiometabolic conditions to gut microbial activities to short-term food consumption [3]. Importantly, urine reflects kidney function, and therefore, urinary metabolomics is a particularly attractive tool in the study of kidney disease [5]. However, so far, these studies have commonly been limited to case–control studies of modest size [7] or focused on a small number of metabolites quantified. A lack of analytical tools to robustly quantify a broad panel of urinary metabolites in thousands of individuals (at low cost) has meant that the regular use of metabolites in large population health studies is not feasible. Recently, developments in NMR have enabled high-throughput metabolomic profiling of urine from large cohorts [3].

Such recent advances also create enhanced opportunities to identify biomarkers that are related to the progression of diabetic nephropathy. Albuminuria is a strong predictor of progression, but, especially at the early stages, the predictive ability is limited as only around one third of individuals with microalbuminuria experienced a progressive decline in kidney function [8]. In addition, eGFR, a widely used surrogate marker to assess kidney filtration capacity, is also less suitable at the early stages of diabetic nephropathy owing to hyperfiltration [9], and in general, the prediction of future eGFR is challenging at these early stages [10]. Therefore, there is a need for novel, easily measurable biomarkers.

This study used urine NMR metabolomic profiling in a large cohort of prospectively followed individuals with type 1 diabetes to identify metabolites associated with the risk of progression of diabetic nephropathy. This knowledge could broaden our understanding of the pathogenesis of diabetic nephropathy.

## Methods

In this study, we included 2670 individuals with type 1 diabetes from the Finnish Diabetic Nephropathy (FinnDiane) study which is an ongoing, nationwide, multicentre study of adults with established type 1 diabetes at the time of enrolment. A full description of the cohort can be found elsewhere [11, 12]. Type 1 diabetes was defined as age of diabetes onset below 40 years and requiring insulin within the first year of diagnosis. Baseline urinary AER was stratified such that normoalbuminuria was defined by an AER below 30 mg/24 h, microalbuminuria by an AER of 30 to 300 mg/24 h and macroalbuminuria by an AER above 300 mg/24 h. This classification was based on the agreement of at least two out of three consecutive urine collections. The presence of end-stage kidney disease (ESKD) was defined as the individual undergoing dialysis or having received a kidney transplant. We estimated eGFR with the Chronic Kidney Disease Epidemiology Collaboration (CKD-EPI) equation [13]. At the baseline study visit, the individuals had provided a 24-h urine sample. We excluded individuals without a baseline 24-h urine sample analysed by NMR, those with no baseline or follow-up information regarding albuminuria and those whose recorded baseline measurement of AER contradicted their consensus classification (from two out of three measurements) into normoalbuminuria, microalbuminuria or macroalbuminuria. We also excluded individuals with ESKD at baseline, but excepting ESKD, we did not further exclude individuals based on baseline eGFR. Medication use was self-reported. Every participant gave written informed consent. The FinnDiane study was approved by the ethics committee of the Helsinki and Uusimaa Hospital District and conducted in accordance with the Helsinki Declaration.

The primary outcome measure was overall progression of diabetic nephropathy defined as an increase in AER to a higher albuminuria category or being diagnosed with ESKD (355 events (13%), 24,145 person-years). Individuals were followed for a mean of 9.0 years (SD 5.0 years) until their first progression event or the latest albuminuria assessment if they did not progress. Additionally, we investigated two subsets: progression from normoalbuminuria (incident diabetic nephropathy) by limiting the participants to those 1999 individuals with normoalbuminuria at baseline (138 events (7%), 18,655 person-years); and progression from macroalbuminuria by limiting the participants to those 347 individuals with macroalbuminuria at baseline (159 events (46%), 2613 person-years).

Metabolite quantification of the baseline urine samples was performed using a proprietary NMR metabolite profiling service (Nightingale Health, Helsinki, Finland). The NMR-based measurements were conducted from 500 μl of stored 24-h urine samples using a 600 MHz Bruker AVANCE III HD NMR spectrometer (Bruker BioSpin, Switzerland) with automated sample changer and cryoprobe. The spectral data were acquired using standard water-suppressed acquisition settings as described in further details in Electronic supplementary material (ESM) 1. The urine samples had been stored at −20 °C for a median time of 17.8 years (IQR 16.3, 19.7 years) prior to the NMR measurements. The analysis included 54 urinary metabolites quantified in absolute concentration units, as well as the simultaneous quantification of urinary creatinine to enable the analysis of metabolite-to-creatinine ratios. For our main analyses, three metabolites with over 50% missingness were omitted (creatine, mannitol and taurine). Missingness indicates that a given metabolite quantification was unsuccessful for a given individual. The missingness is at random to the best of our knowledge having no reason to expect the missingness to be related to the disease outcome status in our prospective analyses. For our univariate statistical analyses, the varying degree of missingness had very limited influence on power. Therefore, we did not impute missing values.

A pre-analytical quality analysis of the influence of storage temperature and storage time (ESM 2 including ESM Figs 1–4 and ESM Tables 1 and 2) on the NMR progression analysis in nine split-aliquot urine samples stored at −20 °C and −80 °C for a median storage time of 1.8 years did not find any significant differences. When comparing creatinine measured in the same sample once by clinical chemistry directly after the sample collection and once by NMR after storage, we found a strong correlation between the two measurements, and differences can be largely attributed to storage effects over a median storage time of 17.8 years.

### Statistical analyses

In a pre-processing step, metabolite concentrations below the detection limit were set to the smallest detected concentrations for each metabolite. We report absolute baseline concentrations for overall progressors and non-progressors (ESM Table 3). All urinary metabolite concentrations were divided by urinary creatinine to normalise for urine volume (ESM Table 4). All analyses and results, apart from ESM Table 3 and the analyses on effect of storage time and temperatures, are based on metabolite-to-creatinine ratios; thus, the term metabolite refers to its metabolite-to-creatinine ratio, if not otherwise stated explicitly. The distributions of these ratios were skewed for all metabolites; therefore, those ratios were further log-transformed. To facilitate comparison of results, all HRs are reported per SD units.

For comparison of baseline clinical characteristics between overall progressors and non-progressors, *p* values were calculated with 10,000 permutations (of the progressor/non-progressor labels), and 95% CIs were estimated with bootstrapping and 10,000 iterations.

Separately for each urine metabolite, time-to-event analyses were performed using Cox proportional hazard regression models [14] on top of baseline albuminuria category and eGFR. Further covariates were sex, calendar year of diabetes diagnosis, baseline age and HbA_1c_. Prior to the analyses, the proportional hazard assumption was tested with Schoenfeld residuals. To cope with violations of the proportional hazard assumption, all Cox models were stratified according to sex, baseline albuminuria category and chronic kidney disease (CKD) stage derived from baseline eGFR. For the same reason, in all models, HbA_1c_ was dichotomised (HbA_1c_> 58.5 mmol/mol or 7.5%). If the proportional hazard assumption was violated additionally by the explanatory metabolite variable, the follow-up time was stratified into distinct intervals following the method of Zhang et al. [15]. This (overall) analysis was repeated with a subset of individuals with normoalbuminuria at baseline and with those with macroalbuminuria.

The *p* value threshold after adjusting for multiple testing was 0.001. Nominal significance refers to a *p* value below 0.05 but above 0.001. Analyses were conducted using R (version 3.6; https://www.r-project.org).

## Results

Among the 2670 individuals with type 1 diabetes, there were 355 (13%) who progressed to either a worse albuminuria category or ESKD, here denoted as overall progressors. The baseline characteristics of the progressors differed from the 2315 non-progressors (Table 1).

**Table 1:** Baseline characteristics of non-progressors and progressors (overall progression)

Characteristic	Non-progressors *n* = 2315	(Overall) Progressors *n* = 355	*p* value
Sex (men %)	49.7 (47.7, 51.7)	57.8 (52.7, 62.8)	0.005
Age (years)	35.7 (34.9, 36.3)	39.3 (37.5, 40.6)	<0.001
Age at diabetes onset (years)	15.4 (14.8, 16.0)	13.0 (11.8, 13.8)	0.001
Diabetes duration (years)	18.5 (17.9, 19.1)	24.2 (22.4, 25.6)	<0.001
HbA_1c_ (mmol/mol)	65.0 (65.0, 66.1)	76.0 (74.3, 78.1)	<0.001
HbA_1c_ (%)	8.1 (8.1, 8.2)	9.1 (8.9, 9.3)	<0.001
Systolic BP (mmHg)	130 (129, 130)	138 (136, 141)	<0.001
Diastolic BP (mmHg)	79 (78, 79)	82 (81, 83)	<0.001
BMI (kg/m^2^)	24.7 (24.5, 24.8)	24.9 (24.6, 25.3)	0.18
Obesity^a^ (%)	8.0 (6.9, 9.1)	14.7 (11.1, 18.5)	<0.001
Total cholesterol (mmol/l)	4.73 (4.69, 4.77)	5.10 (4.97, 5.19)	<0.001
HDL-cholesterol (mmol/l)	1.35 (1.33, 1.36)	1.21 (1.17, 1.25)	<0.001
Triacylglycerol (mmol/l)	0.94 (0.92, 0.96)	1.35 (1.26, 1.47)	<0.001
eGFR (ml min^−1^ [1.73 m]^−2^)	104 (103, 105)	81 (71, 89)	<0.001
Normoalbuminuria (%)	80.4 (78.8, 82.0)	38.9 (33.8, 43.9)	<0.001
Microalbuminuria (%)	11.5 (10.2, 12.8)	16.3 (12.7, 20.3)	0.01
Macroalbuminuria (%)	8.1 (7.0, 9.2)	44.8 (39.4, 49.9)	<0.001
Lipid-lowering medication (%)	7.7 (6.7, 8.8)	24.1 (19.8, 28.7)	<0.001
Anti-hypertensive medication			
RAAS inhibitors (%)	22.3 (20.6, 24.0)	56.0 (50.7, 61.3)	<0.001
Calcium channel blockers (%)	5.5 (4.6, 6.4)	23.8 (19.5, 28.3)	<0.001
β blockers (%)	5.9 (5.0, 7.0)	27.0 (22.4, 31.6)	<0.001
Diuretics (%)	5.3 (4.4, 6.3)	30.9 (26.3, 35.7)	<0.001
Others (%)	0.3 (0.1, 0.6)	1.7 (0.6, 3.1)	0.003

Clinical characteristics are represented by medians with 95% CIs or percentages with 95% CIs for binary variables

^a^Obesity was defined as a BMI above 30 kg/m^2^

*p* values were calculated from 10,000 permutations and the CIs with bootstrapping from 10,000 iterations

RAAS inhibitors, renin–angiotensin–aldosterone system inhibitors including ACE inhibitors and angiotensin II receptor blockers

There were significant differences between progressors and non-progressors for 17 metabolites (ESM Table 4). Progressors showed an increased ratio (*p* < 0.0001) for 2-hydroxyisobutyrate, dimethylamine, isoleucine and pseudouridine and a decreased ratio for 3-hydroxyisobutyrate, 3-hydroxyisovalerate, citrate, 4-deoxyerythronic acid, ethanolamine, glycine, glycolic acid, histidine, hypoxanthine, indoxyl sulphate, 1-methylnicotinamide, trans-aconitate and tyrosine.

**Fig. 1:**
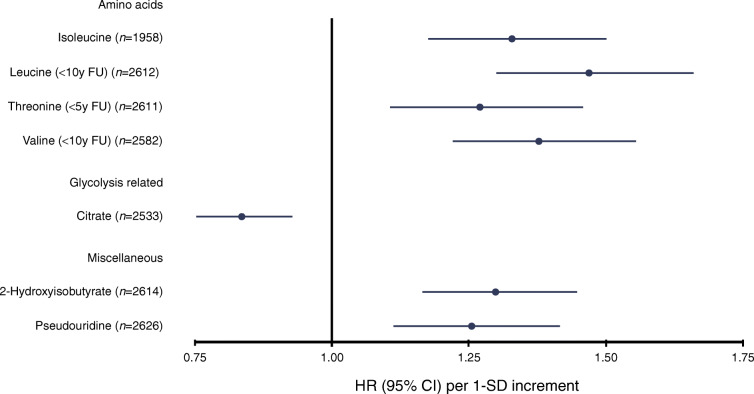
Standardised HRs and 95% CIs for urinary metabolites that were significantly associated with incidence of (overall) progression after accounting for multiple testing (*p* < 0.001) in all 2670 individuals. Urine metabolites were scaled to creatinine and log-transformed. The analysis was adjusted for sex and baseline age, year of diabetes diagnosis, baseline glycaemic control (HbA_1c_ > 58.5 mmol/mol or 7.5%) and baseline CKD stage and albuminuria class. HRs were scaled to SD units. The proportional hazard assumption was tested with Schoenfeld residuals, and follow-up times were split when violated. FU, follow-up; y, year

Assessing the incidence of progression with Cox models, seven urinary metabolites showed a significant association with overall progression after adjustment for sex, age and diabetes duration as well as baseline glycaemic control, albuminuria and CKD stages (Fig. 1). For leucine, valine and threonine, the association with progression was significant early on in the follow-up period but did not remain significant after 10 years for leucine and valine or 5 years of follow-up for threonine. Leucine (until 10 years of follow-up) showed the strongest association of all urinary metabolites with overall progression with an HR of 1.47 (95% CI 1.30, 1.66, *p* = 6.83 × 10^−10^). In comparison, this HR was lower than that for insufficient glycaemic control in individuals with normoalbuminuria (2.41 [1.54, 3.76], *p* = 0.0001) in an equally adjusted model and comparable with the HR for sex (1.34 [0.95, 1.90], *p* = 0.09) from the same model, albeit the latter one was not statistically significant. Valine until 10 years of follow-up (1.38 [1.22, 1.56], *p* = 2.17 × 10^−7^), isoleucine (1.33 [1.18, 1.50], *p* = 5.19 × 10^−6^), 2-hydroxyisobutyrate (1.30 [1.16, 1.45], *p* = 2.40 × 10^−6^), threonine until 5 years of follow-up (1.27 [1.11, 1.46], *p* = 0.0007) and pseudouridine (1.25 [1.11, 1.42], *p* = 0.0002) were also associated with progression of diabetic nephropathy. The relationship was inverse for urinary citrate (0.84 [0.75, 0.93], *p* = 0.0008). The complete results can be found in ESM Fig. 5 and ESM Table 5 to support future studies, in accordance with recommended reporting practices for NMR metabolomic profiling studies [16].

Regarding progression from normoalbuminuria only (1999 individuals, 138 events), 2-hydroxyisobutyrate was significantly associated with incident diabetic nephropathy when adjusted for all covariates including baseline glycaemic control, albuminuria and CKD stage with an HR of 1.56 [1.25, 1.95] (*p* = 9.58 × 10^−5^). This metabolite was also associated with overall progression. No other metabolite showed a significant association with progression after taking multiple testing into account (ESM Fig. 5, ESM Table 6).

**Fig. 2:**
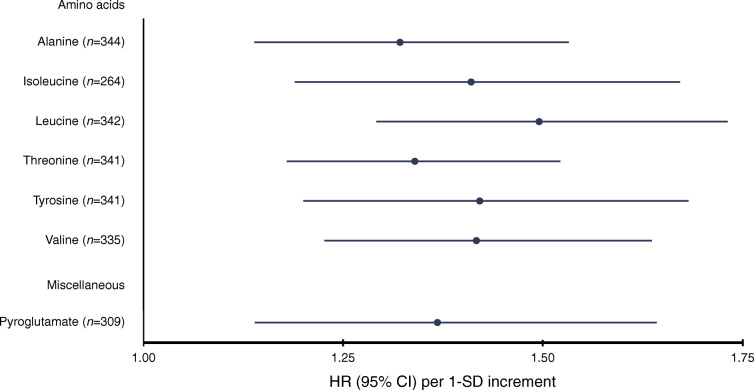
Standardised HRs and 95% CIs for urinary metabolites that were significantly associated with incidence of progression to ESKD after accounting for multiple testing (*p* < 0.001) in 347 individuals with macroalbuminuria. Urine metabolites were scaled to creatinine and log-transformed. The analysis was adjusted for sex and baseline age, year of diabetes diagnosis, baseline glycaemic control (HbA_1c_ > 58.5 mmol/mol or 7.5%) and baseline CKD stage and albuminuria class. HRs were scaled to SD units. The proportional hazard assumption was tested with Schoenfeld residuals, and follow-up times were split when violated

To identify urine metabolites that are particularly indicative of progression to the most severe and costly stages of diabetic nephropathy, we further examined associations with the progression from macroalbuminuria to ESKD (347 individuals, 159 events). Six amino acids and one derivate of an amino acid were associated with the incidence of ESKD when adjusted for all covariates including baseline glycaemic control, albuminuria and CKD stage (Fig. 2). As in the overall results, leucine (1.50 [1.29, 1.73], *p* = 7.50 × 10^−8^), valine (1.42 [1.23, 1.64], *p* = 2.20 × 10^−6^), isoleucine (1.41 [1.19, 1.67], *p* = 7.61 × 10^−5^) and threonine (1.34 [1.18, 1.52], *p* = 6.99 × 10^−6^) were associated with progression. In addition, tyrosine (1.42 [1.20, 1.68], *p* = 4.55 × 10^−5^), alanine (1.32 [1.14, 1.53], *p* = 0.0002) and pyroglutamate (1.37 [1.14, 1.64], *p* = 0.0008) were also associated with incident ESKD. Alanine (up to 5 years of follow-up) was also nominally associated with overall progression (1.24 [1.07, 1.43], *p* = 0.004). The complete results for progression from macroalbuminuria to ESKD can be found in ESM Fig. 5 and ESM Table 7. When excluding those individuals with macroalbuminuria in CKD stage 5, the results persisted (see ESM Fig. 6).

Glycine and threonine showed opposing associations when looking at individuals with normoalbuminuria and macroalbuminuria separately. However, most of these associations were only nominally significant. Higher glycine (1.17 [1.02, 1.33], *p* = 0.02) and threonine (1.34 [1.18, 1.52], *p* = 6.99 × 10^−6^) were associated with incident ESKD in those with macroalbuminuria, but lower glycine (0.80 [0.67, 0.95], *p* = 0.01) and threonine (0.81 [0.67, 0.98], *p* = 0.03) were associated with progression from normoalbuminuria (incident diabetic nephropathy).

## Discussion

In this study, we employed a high-throughput NMR platform to analyse 51 metabolite-to-creatinine ratios from 24-h urine samples of 2670 individuals with type 1 diabetes and found that ten ratios were associated with progression of diabetic nephropathy after adjusting for baseline albuminuria category and CKD stage. Importantly, there were differences between the urinary profile for overall progression and progression from normo- and macroalbuminuria suggesting 2-hydroxyisobutyrate as a potential marker for progression from normoalbuminuria (i.e. new-onset diabetic nephropathy) and branched-chain amino acids as markers for overall progression and progression from macroalbuminuria to ESKD.

Several metabolites point towards a link between insulin resistance and progression of diabetic nephropathy. Urinary amino acid and in particular branched-chain amino acids were associated with overall progression as well as progression from macroalbuminuria to ESKD. These novel findings are noteworthy in the context of the many studies on plasma concentrations of branched-chain amino acids in the field of type 2 diabetes. The first study to show associations between plasma levels of branched-chain amino acids with obesity and insulin resistance dates back 50 years [17]. These amino acids also represent the most consistent biomarkers identified by blood metabolomics to be predictive of future type 2 diabetes risk [18]. In physiological studies and human genetics, there is even evidence that links higher levels of insulin resistance causally to higher concentrations of circulating branched-chain amino acids [19]. Furthermore, insulin resistance has been observed in individuals with type 1 diabetes and linked to the increasing prevalence of obesity in type 1 diabetes [20], and also in our cohort, progressors were more likely to be obese at baseline. Importantly, it has been shown in type 1 diabetes that insulin resistance precedes microalbuminuria [21]. A previous, small study of 200 individuals from FinnDiane [22] found that serum metabolites that have been associated with new-onset albuminuria have also been associated with insulin resistance. Urine concentrations of branch-chain amino acids may depend on protein intake. We do not have data on diet, but secretion for most amino acids is not strongly influenced by protein intake in healthy individuals [23]. However, high protein intake in prevalent CKD may further kidney function decline [24]. Reduced tubular reabsorption may play an important role. A recent review [25] reported that the expression of amino acid transporter *Slc6a19*, which has a higher affinity to neutral amino acids than branched-chain amino acids, was increased in animal models of diabetes. In line with reduced reabsorption, a study on type 2 diabetes found that individuals who progressed to ESKD had lower serum concentrations of leucine and valine [26].

Urinary 2-hydroxyisobutyrate is another metabolite that links progression of diabetic nephropathy with insulin resistance. It derives from the degeneration of proteins by gut microbiota [27]. It was shown that urinary 2-hydroxyisobutyrate was higher in obese, insulin-resistant men compared with age-matched, lean control participants [27]. In our cohort, 2-hydroxyisobutyrate was associated with overall progression, the progression from normoalbuminuria and nominally associated with the progression from macroalbuminuria to ESKD. The fact that 2-hydroxyisobutyrate is associated with both progressions is important as progression from normoalbuminuria is related to changes in albumin excretion, and progression from macroalbuminuria is more related to a drop in filtration capacity. A study found no correlation between eGFR and serum 2-hydroxyisobutyrate in non-diabetic individuals with CKD stages 3 and 4 [28] and reported higher serum concentrations of 2-hydroxyisobutyrate compared with healthy control participants, suggesting that renal clearance of the metabolite is mainly dependent on active tubular transport. Moreover, clinically relevant concentrations of 2-hydroxyisobutyrate given to a human renal proximal tubule cell line resulted in higher expression of mesenchymal markers and loss of epithelial features without affecting its mitochondrial activity [28]. Mitochondrial homeostasis is very important for the functioning of reabsorption [29]. Therefore, fitting with our observation of 2-hydroxyisobutyrate as an early marker of progression, there is some evidence that the higher concentrations in urine in individuals with normoalbuminuria might not result from reduced tubular reabsorption but from higher serum concentrations. In contrast to 2-hydroxyisobutyrate, the branched-chain amino acids did not show any association with progression from normoalbuminuria but a strong association with progression from macroalbuminuria, pointing more towards reduced tubular reabsorption.

Urinary pseudouridine has been discussed as a potential glomerular filtration marker, and in our study, it was associated with overall progression. Previous studies in serum have shown elevated concentrations of pseudouridine in renal failure and uraemia, and the concentrations were associated with CKD [30]. In type 1 diabetes, serum pseudouridine has been shown to be associated with eGFR decline in individuals with proteinuria in CKD stage 3 [31]. Pseudouridine-to-creatinine ratios measured in spot urine were slightly decreased in individuals with CKD, but it is unclear whether these cross-sectional differences were statistically significant [32]. Previously, pseudouridine was dismissed as a glomerular filtration marker due to tubular reabsorption. However, newer findings have shown that pseudouridine and eGFR are correlated, and while the calculation of eGFR has to take sex into account, pseudouridine excretion was shown to be less dependent on sex [30]. Therefore, our results suggest that pseudouridine is a glomerular filtration marker.

In addition to filtration, several urinary metabolites are linked to renal tubular damage or protection. Low urinary citrate was associated with overall progression. This observation is in line with a study showing that administration of citrate salts reduces tubulointerstitial injury and slows eGFR decline [33]. Additionally, metabolic acidosis in individuals with CKD was associated with lower urinary citrate [34]. In general, our results on citrate are in accordance with a previous small cross-sectional study on CKD [34] associating urinary citrate with tubular protection.

Previous studies investigating tubular damage showed links with urinary glycine, alanine and pyroglutamate. In our cohort, we observed differing associations for progression for urinary glycine in individuals with normo- and macroalbuminuria. Lower glycine in urine was nominally associated with progression from normoalbuminuria and higher glycine with progression from macroalbuminuria. Urinary threonine followed the same directions of association, and there are several degradation processes for threonine, one leading to the synthesis of glycine [35]. Low urinary glycine has previously been associated with incident eGFR reduction below 60 ml min^−1^ [1.73 m]^−2^ in the case–control matched Framingham Offspring cohort of 386 individuals [36]. This setting is more comparable to our subanalysis in individuals with normoalbuminuria with a mean eGFR of 105 ml min^−1^ [1.73 m]^−2^ at baseline. As in the Framingham Offspring cohort, low urinary glycine was nominally associated with progression from normoalbuminuria. In animal models, dietary glycine has been shown to protect against cyclosporin-mediated proximal tubular damage [36]; such damage may occur early in kidney disease and may even precede glomerular changes [37]. We have previously shown that genetic variants in the *GLRA3* gene encoding a glycine receptor are associated with albuminuria [38]. Interestingly, in our cohort, the association changed when looking at the progression from macroalbuminuria. In this scenario, higher and not lower urinary glycine was associated with progression. The reabsorption of glycine through the kidney plays an important role for the bioavailability of glycine [39] and is facilitated to a similar extent by energy-dependent transport and non-energy-dependent transport [40]. Lower urinary glycine in those individuals who progressed from normoalbuminuria might therefore be related to lower serum concentrations. On the other hand, the higher concentrations in progressors to ESKD point towards a reduced rate of energy-dependent reabsorption. Furthermore, increased concentrations of glycine and alanine were found in rat urine after inducing damage to the proximal tubule [41] reflecting that alanine and glycine energy-dependent reabsorption is mediated by neutral amino acid transporters such as *SLC6A19* [40] and both were associated with incident ESKD in this study. Urinary pyroglutamate, which was associated with progression from macroalbuminuria to ESKD, has previously been linked to incident macroalbuminuria in individuals with type 2 diabetes and microalbuminuria [42]. A previous study in type 1 diabetes with 25 individuals linked pyroglutamate to the progression of early kidney disease from normoalbuminuria to microalbuminuria [43]. However, in our study, we did not find any link between pyroglutamate and such early progression. We suggest that, in a healthier kidney, pyroglutamate is efficiently reabsorbed [44], and with worsening kidney disease, the reabsorption capabilities diminish.

Finally, we also found urinary markers that have been previously linked to responses to oxidative stress and hypoxia, such as tyrosine. Oxidative stress is a hallmark of CKD [45], and the interplay between oxidative stress and hypoxia critically contributes to kidney injury [46]. In our study, higher urinary tyrosine was associated with progression to ESKD in individuals with macroalbuminuria. An inverse association was shown in a previous study in type 2 diabetes that only investigated the earlier stages of kidney disease in individuals who had either normo- or microalbuminuria at baseline [47]. These observations may be influenced by oxidative stress [45]. Molnár et al. suggested that both the healthy and the damaged kidney retain para-tyrosine that is converted from phenylalanine. In the presence of free radicals, phenylalanine and consequently tyrosine are hydroxylated in para, meta and ortho positions, and the authors observed that the ortho-tyrosine excretion was enhanced in type 2 diabetes through an increased tubular secretion and production [45]. Therefore, we would indeed expect increased concentrations of urinary tyrosine in the presence of increased oxidative stress. A previous study on urine metabolites in diabetes also found links to mitochondrial activity [7]. In further concordance with that study, we found the same direction of associations for 3-hydroxyisovalerate and glycolic acid in the overall and macroalbuminuria analysis.

There are some limitations of our study. The associations between urine metabolites and progression do not allow for any causal conclusions. Furthermore, the results have not as yet been replicated in an independent cohort as we did not have access to other large cohorts with type 1 diabetes, 24-h urine collections and long-term follow-up. Large-scale urinary NMR analysis, such as this study, will shortly be taken up by many cohorts. In the future, it will probably be possible to validate these results with other large cohorts, and therefore, these biomarkers will potentially go beyond providing disease aetiology information to disease prediction. These univariate analyses did not consider the correlation between metabolites (ESM Fig. 7), and future studies will need to investigate which signals are independent. In addition, we do not have dietary data to assess the influence of diet. We cannot exclude an effect of storage time; however, sensitivity analyses for creatinine measured by clinical chemistry at sampling time did not find a change in the direction of the associations. The study also has several strengths starting with the size of the cohort. In addition, the urine was measured over 24 h, which is the gold standard of urine collections. However, the applicability of the results to morning or spot urine collections remains to be addressed. The FinnDiane cohort is thoroughly characterised and has a long follow-up period.

In summary, this study found that ten out of 51 urinary metabolites were associated with progression of diabetic nephropathy even after adjusting for baseline albuminuria category and CKD stage. We found differences between overall progression and progression from normo- and macroalbuminuria such as 2-hydroxyisobutyrate as a potential marker for progression from normoalbuminuria. Amino acids and, in particular, the branched-chain amino acids were strongly associated with progression and especially with the progression from macroalbuminuria to ESKD. These results provide new potential urinary biomarkers that were associated with progression beyond the traditional markers of albuminuria and estimated kidney filtration rates. This study highlights the potential of routinely analysing urinary metabolites on a larger scale as urinary NMR metabolomic profiling is a reliable high-throughput method.

## Supplementary Information

ESM 1 (PDF 1.53 kb)

ESM 2 (XLSX 33 kb)

## Data Availability

Aggregated summary data are made available in the ESM. Individual-level data cannot be shared for reasons of patient privacy.

## Abbreviations

CKD: Chronic kidney disease
ESKD: End-stage kidney disease
FinnDiane: Finnish Diabetic Nephropathy study
