# Supplementary material for: Urinary metabolite profiling and risk of progression of diabetic nephropathy in 2670 individuals with type 1 diabetes
Source: Diabetologia. 2021 Oct 22;65(1):140–9. doi: 10.1007/s00125-021-05584-3 (PMC8660744; doi:10.1007/s00125-021-05584-3)
Supplement: Supplementary file 1 — (PDF 1.53 kb) [file 125_2021_5584_MOESM1_ESM.pdf]

ESM:

Urinary metabolite profiling and risk of progression of diabetic nephropathy in 2,670 individuals with type 1 diabetes

Stefan Mutter [1,2,3], Erkka Valo [1,2,3], Viljami Aittomäki [4], Kristian Nybo [4], Lassi Raivonen [4], Lena M Thorn [1,2,3,5], Carol Forsblom [1,2,3], Niina Sandholm [1,2,3], Peter Würtz [4], Per-Henrik Groop [1,2,3,6*]

[1]Folkhälsan Institute of Genetics, Folkhälsan Research Center, Helsinki, Finland

[2]Department of Nephrology, University of Helsinki and Helsinki University Hospital, Helsinki, Finland

[3]Research Program for Clinical and Molecular Metabolism, Faculty of Medicine, University of Helsinki, Helsinki, Finland

[4]Nightingale Health Ltd, Helsinki, Finland

[5]Department of General Practice and Primary Health Care, University of Helsinki and Helsinki University Hospital, Helsinki, Finland

[6]Department of Diabetes, Central Clinical School, Monash University, Melbourne, Victoria, Australia

TABLE OF CONTENTS

Table of Contents 2

ESM 1: Urinary metabolite profiling by high-throughput NMR spectroscopy 3

ESM 2: Storage temperature and storage time analysis 6

Methods 6
Results 6
Conclusions 8

ESM 3: ESM table legends 9

ESM 4: ESM figures 11

ESM 5: FinnDiane study centers 21

List of abbreviations in ESM Figure 1 23

ESM References 25

ESM 1: URINARY METABOLITE PROFILING BY HIGH-THROUGHPUT NMR SPECTROSCOPY

Metabolic profiling of urine in large biobanked sample collections can enable numerous research applications [1–4]. Here we used established NMR spectroscopy protocols and an automated total-line-shape fitting method to quantify up to 54 urine biomarkers in 24 hours urine samples from 2,670 participants in the prospective FinnDiane study. This method is based on a high-throughput nuclear magnetic resonance metabolomics approach as widely used previously for serum and plasma samples [5]. The automated analyses provide absolute concentrations of 54 urine metabolites and their ratio to creatinine (ESM Table 3-4).

The sample preparation and NMR acquisition parameters were designed for high-throughput rather than optimising for widest possible metabolite coverage. Therefore, metabolites were initially selected based on feasibility for automated quantification in high-throughput NMR spectroscopy. This approach emphasises metabolites at high abundance in urine, and those which generate minimal signal overlap in the proton NMR spectrum. As such, the metabolite selection was not based on prior biological relevance of the selected metabolites or emphasis of certain metabolic pathways. Representative proton NMR spectra and metabolite assignment can be found in for example in Tynkkynen et al [6]. Briefly the actual NMR procedure went as follows, urine samples are thawed overnight and subsequently centrifuged (5 min, 3000g). Robotic liquid handlers (JANUS 8-tip workstation; PerkinElmer Inc, USA) are used to mix 70 µL of phosphate buffer (1.5 M K_2HPO_4 and 1.5 M NaH_2PO_4 in D_2O, pH 7.0; including 5.8 mM sodium 3-(trimethylsilyl)propionate-2,2,3,3-d_4 and 30.8 mM sodium azide; [7]) and 450 µL urine into 5mm NMR tubes placed on 96-tube racks. For each 96-tube rack, two quality

control samples of stock urine are included to enable tracking of consistency of the NMR measurements over time. A 600 MHz Bruker AVANCE IIIHD NMR spectrometer with automated SampleJet sample changer and cryoprobe (CryoProbe Prodigy TCI) is used to acquire the spectral data at 298K. The spectroscopy settings are standard water-suppressed measurements (Bruker noesypresat pulse sequence with mixing time of 10 ms and irradiation field of 25 Hz) using 32 scans per sample with 5.1 s recycle time. A metabolite quantification protocol based on automated implementation of total-line-shape fitting [8] is applied after piece-wise spectral alignment. The scientific literature on chemical shifts and J-couplings for high-abundance metabolites in urine was used to assign metabolite peak shapes [2, 9]. The metabolite identifications have further been confirmed with spiking and concentrations calibrated using standard addition.

The high-throughput metabolite quantification offered by the targeted Nightingale NMR platform offers some pros and cons in comparison to more widely used approaches based on mass spectrometry for metabolite quantification. The following points are worthwhile considerations in this respect:

1) the automated NMR-based approach offers the possibility to measure large sample collections at modest cost, compared to the price of mass spectometry assays,
2) the sample preparation is minimal with the NMR based approach, since only buffer is added to the sample and no extraction step is needed that could cause drift in the measurements,
3) the consistency of the NMR-based measurements over time is very robust, which is favourable for measurements of large urine sample collections,
4) the metabolite quantification is provided in absolute concentrations rather than ion count. On the other hand, the metabolite coverage is more limited with NMR compared

to mass spectrometry, since detection of molecules at low concentration is challenging by NMR, especially in a high-throughput setup.

ESM 2: STORAGE TEMPERATURE AND STORAGE TIME ANALYSIS

Methods

We investigated the analytical effects of the urinary metabolite measures in relation to sample storage temperature and consistency of creatinine measured by clinical chemistry at the time of sample collection. In these pre-analytical quality analysis, split-aliquote urine samples for nine individuals (three women, six men) were stored continuously at both −20˚C and −80˚C for a median storage time of 1.8 years (1.8, 2.4 years). Those nine individuals had a median age of 48.4 years (41.9, 53.0 years) and a median duration of diabetes of 36.8 years (32.4, 43.4 years). We excluded seven biomarkers that were missing for at least five individuals from the full set of 54 markers and assessed the effect of storage time with a linear model with storage time as independent and the logarithm of the ratio of the biomarker's absolute concentrations at −20˚C and −80˚C as dependent variable. In this analysis, a β-coefficient of 0 indicates no effect of storage time. Subsequently, we took advantage of the fact that creatinine was measured clinically at the time the samples were taken and again by NMR for this study. We assessed the association between these two measurements in one linear model for all 2,628 samples that had both measures available and in separate linear models per sample year. The p-value threshold for significance after adjusting for multiple testing was 0.001. Nominal significance refers to a p-value below 0.05 but above 0.001.

Results

Storage temperature and storage time did not have a strong influence on the stability of the urine metabolite concentrations over a median follow-up of 1.8 years. When looking at the effect of storing samples at −20˚C and −80˚C, we did not find significant differences after adjusting for multiple testing (ESM Table 1). There were only three metabolites where the differences in the absolute concentrations were nominally significant: 4-deoxythreonate (−0.0023 mmol/L, p = 0.03) and tryptophan (−0.0031 mmol/L, p = 0.03) had slightly lower

concentrations at −80˚C compared to −20˚C. Xanthosine concentrations were slightly elevated at −80˚C compared to −20˚C (0.012 mmol/L, $p = 0.04$). We further investigated the Bland-Altman plots (ESM Figure 1) to test the agreement of the two measurements. We found two samples (sample 1 and sample 7 in the figure) that were responsible for most of the divergence. The agreement between the two measures was high in general and there was a tendency that the samples diverged in the same direction providing for example higher concentrations at −20˚C for formate and lower concentrations for xanthosine. When taking storage time into account, we did not find any significant effect after adjusting for multiple testing (ESM Table 2). But metabolites such as ethanol (β=0.52, p=0.01), indoxyl sulfate (β=0.15, p=0.02) and especially alanine (β=0.17, p=0.0013) showed a nominal effect of the storage temperature and time. This effect for alanine was likely driven by prominent outliers (ESM Figure 2). Notably, we did not observe any significant effect on creatinine (β=0.04, p=0.53). It is important to note that the storage time for this small number of split-aliquote samples was short (median 1.8 years) when compared to the overall storage times of the full set (median 17.8 years).

NMR measurements of urinary creatinine corresponded very well to the urinary creatinine measured by clinical chemistry (β=0.95, r^2=0.77) in our study samples (ESM Figure 3). In this full set, we found a significant effect of storage time (β=0.02, p=1.9 × 10−13) corresponding to a 1.40% underestimation of creatinine per storage year. The median creatinine measured by clinical chemistry at the time of sample collection was 5.30 mmol/L and 4.34 mmol/L by NMR analysis after a median storage time of 17.8 years (-18%). When we repeated the association analysis only including the latest samples starting from the beginning of 2015, the association between creatinine measured by NMR and clinical chemistry was therefore stronger (β = 0.99, r^2=0.88). ESM Figure 4 summarises the association between creatinine measured by NMR and clinical chemistry by year and highlights that the NMR-measured creatinine quantification reproduced the clinical chemistry measurements of creatinine very well.

Conclusions

Urine has been stored at -20˚C prior to the NMR analysis and our quality analysis did not find any significant difference to storage at -80˚C for up to 2 years. When comparing creatinine measured by NMR to the one measured by clinical chemistry, with the latter analysed right after the sample collection, we found a strong correlation between both measurements and differences (-18%) can be largely attributed to storage effects over a median storage time of 17.8 years. As sensitivity analyses, we repeated the main progression analysis with creatinine values adjusted for storage time and additionally with creatinine measured by clinical chemistry. The direction of associations in both sensitivity analyses did not change (data not shown).

ESM 3: ESM TABLE LEGENDS

All ESM tables are provided separately in Excel format. The table legends are as follows:

ESM Table 1. *Mean absolute concentration differences and their standard deviations in urine samples for nine individuals stored continuously at −20˚C and −80˚C for a median storage time of 1.8 year. Data from seven metabolites has been removed due to a high number of missing values. P-values were calculated by pairwise t-test. A p-value below 0.001 denotes statistical significance.*

ESM Table 2. *Effect of storage time on the metabolite ratio at −20˚C and −80˚C with a linear model with storage time as independent and logarithm to the base 2 of the ratio as the dependent variable based on split-aliquote urine samples for nine individuals that were stored for a median of 1.8 years. In this analysis, a β-coefficient of 0 indicates no effect of storage time on the ratio. A p-value below 0.001 denotes statistical significance.*

ESM Table 3. *Metabolites' HMDB IDs and CAS Registry Numbers and baseline median absolute concentrations and 95% confidence intervals of urinary metabolites for non-progressors and progressors (overall progression). P-values were calculated from 10,000 permutations and the confidence intervals with bootstrapping from 10,000 iterations. A p-value below 0.001 denotes statistical significance.*

ESM Table 4. *Baseline metabolite to creatinine ratios: mean and standard deviation and median and 95% confidence interval (CI) for non-progressors and progressors (overall progression). P-values were calculated from 10,000 permutations and the confidence intervals with bootstrapping from 10,000 iterations. A p-value below 0.001 denotes statistical significance.*

ESM Table 5. *Standardised hazard ratios and 95% confidence intervals for all urinary metabolites in the overall Cox regression analysis that assesses progression of diabetic nephropathy for all 2,670 individuals. Urine metabolites were scaled to creatinine and log-transformed. The analysis was adjusted for sex and baseline age, year of diabetes diagnosis, baseline glycemic control (HbA_{1c} > 58.5 mmol/mol or 7.5%) and baseline CKD stage and albuminuria class. Hazard ratios were scaled to SD-*

units. A p-value below 0.001 denotes statistical significance. The proportional hazard assumption was tested with Schoenfeld residuals and follow-up times were split when violated. FU: follow-up; y: years.

ESM Table 6. *Standardised hazard ratios and 95% confidence intervals for all urinary metabolites in the Cox regression analysis that assesses progression of diabetic nephropathy for 1,999 individuals with normoalbuminuria at baseline. Urine metabolites were scaled to creatinine and log-transformed. The analysis was adjusted for sex and baseline age, year of diabetes diagnosis, baseline glycemic control (HbA_{1c} > 58.5 mmol/mol or 7.5%) and baseline CKD stage and albuminuria class. Hazard ratios were scaled to SD-units. A p-value below 0.001 denotes statistical significance. The proportional hazard assumption was tested with Schoenfeld residuals and follow-up times were split when violated. FU: follow-up; y: years.*

ESM Table 7. *Standardised hazard ratios and 95% confidence intervals for all urinary metabolites in the Cox regression analysis that assesses progression to ESKD for 347 individuals with macroalbuminuria. Urine metabolites were scaled to creatinine and log-transformed. The analysis was adjusted for sex and baseline age, year of diabetes diagnosis, baseline glycemic control (HbA_{1c} > 58.5 mmol/mol or 7.5%) and baseline CKD stage and albuminuria class. Hazard ratios were scaled to SD-units. A p-value below 0.001 denotes statistical significance. The proportional hazard assumption was tested with Schoenfeld residuals and follow-up times were split when violated. FU: follow-up; y: years.*

ESM 4: ESM FIGURES

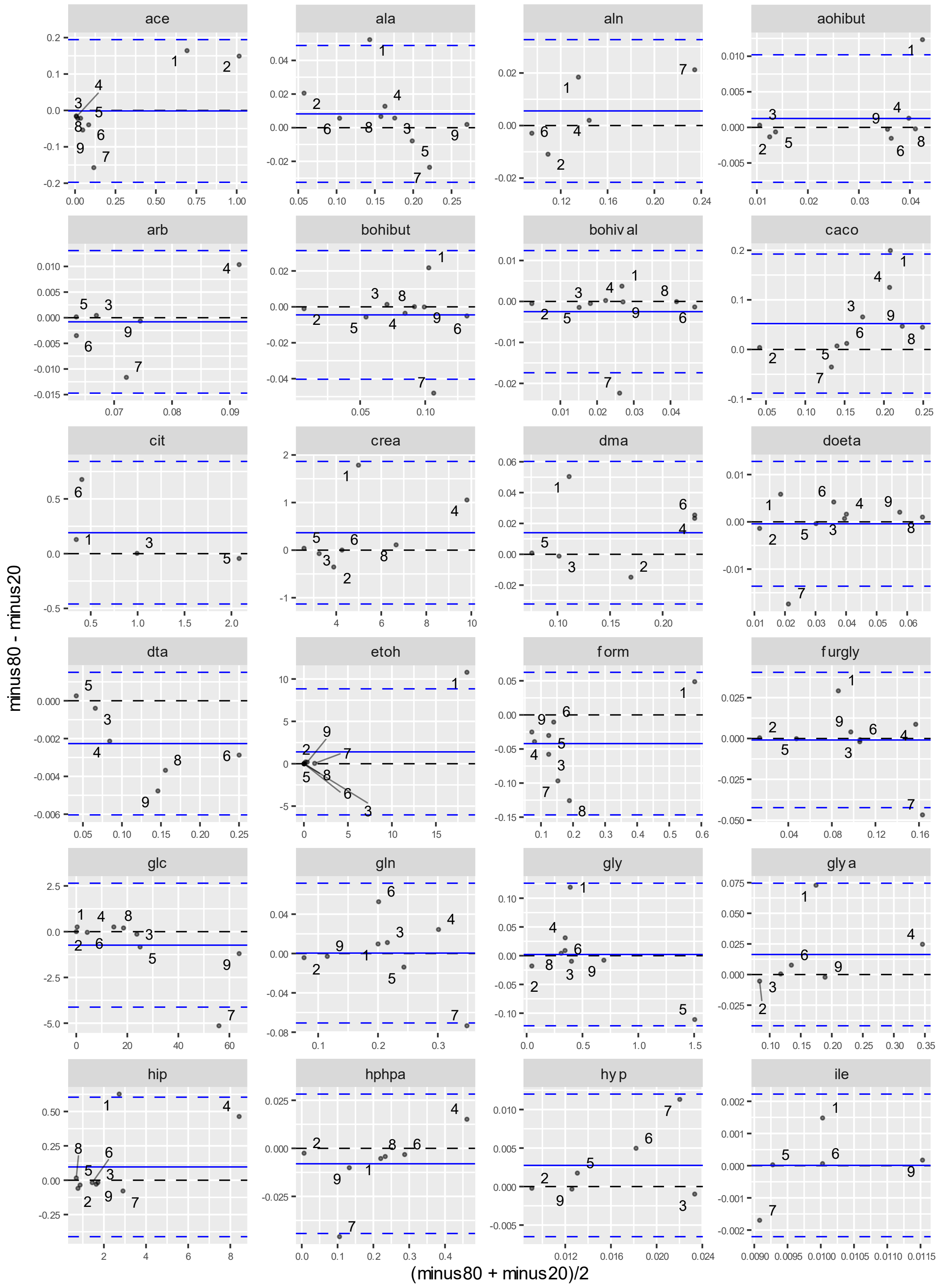

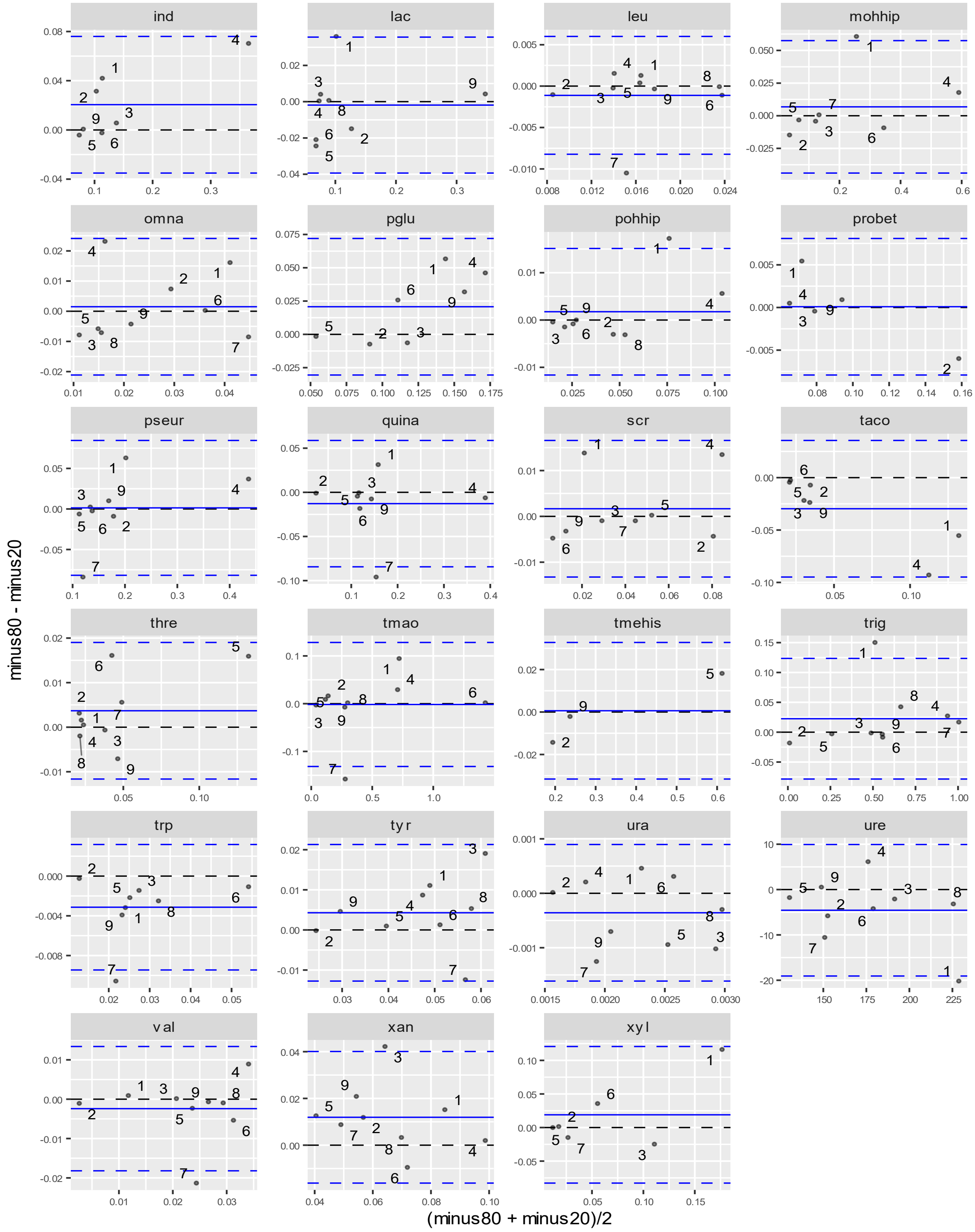

ESM Figure 1. *Bland-Altman plots for nine split-aliquote urine samples from 24h collections stored at −20˚C and −80˚C. The samples are numbered from one to nine and were stored for a median of 1.8 years. The abbreviations for the metabolite names can be found in the list of abbreviations in this ESM (Pages 23/24).*

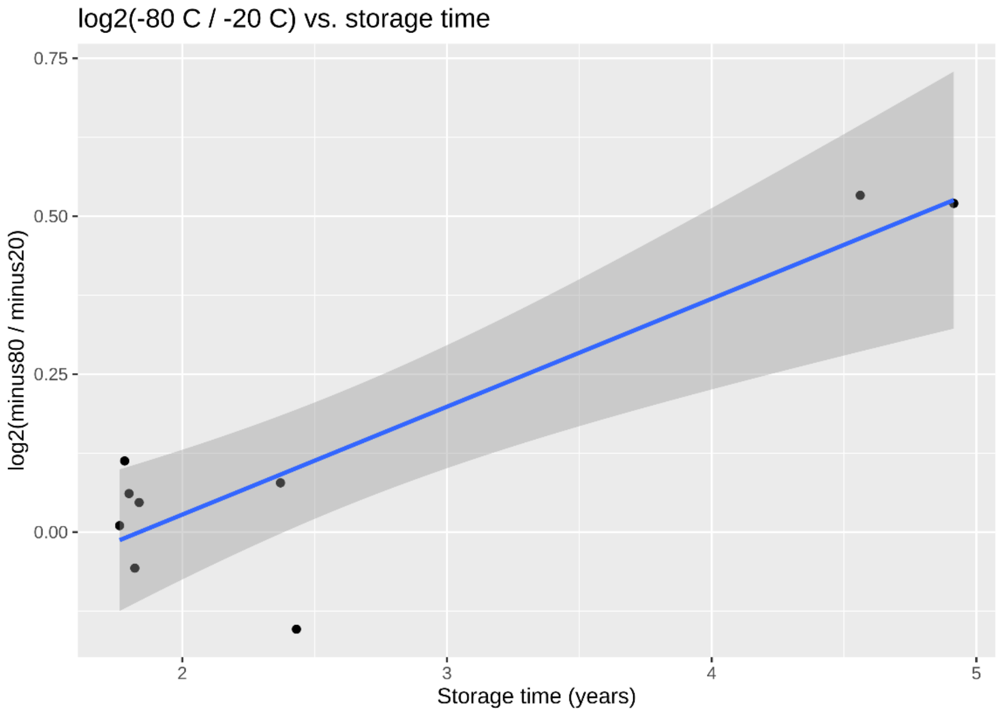

ESM Figure 2. *Relationship of storage time to the logarithm to the base 2 of the metabolite ratio at −20˚C and −80˚C for alanine based on nine split-aliquote urine samples from 24h collections. The samples were stored for a median of 1.8 years.*

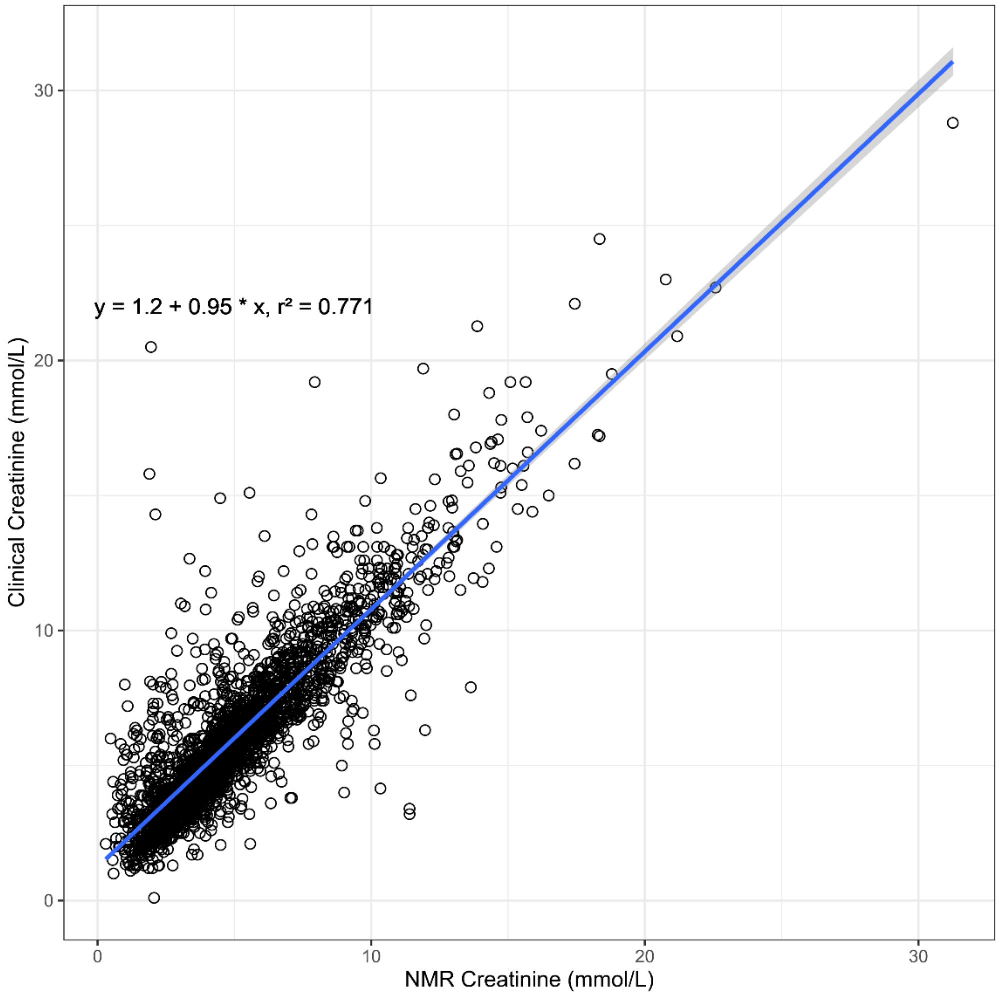

ESM Figure 3. *Correlation of creatinine measured by clinical chemistry at the time of sampling with creatinine measured by NMR after a median storage time of 17.8 years at −20°C for 2,628 urine samples from 24h urine collections where data was available*

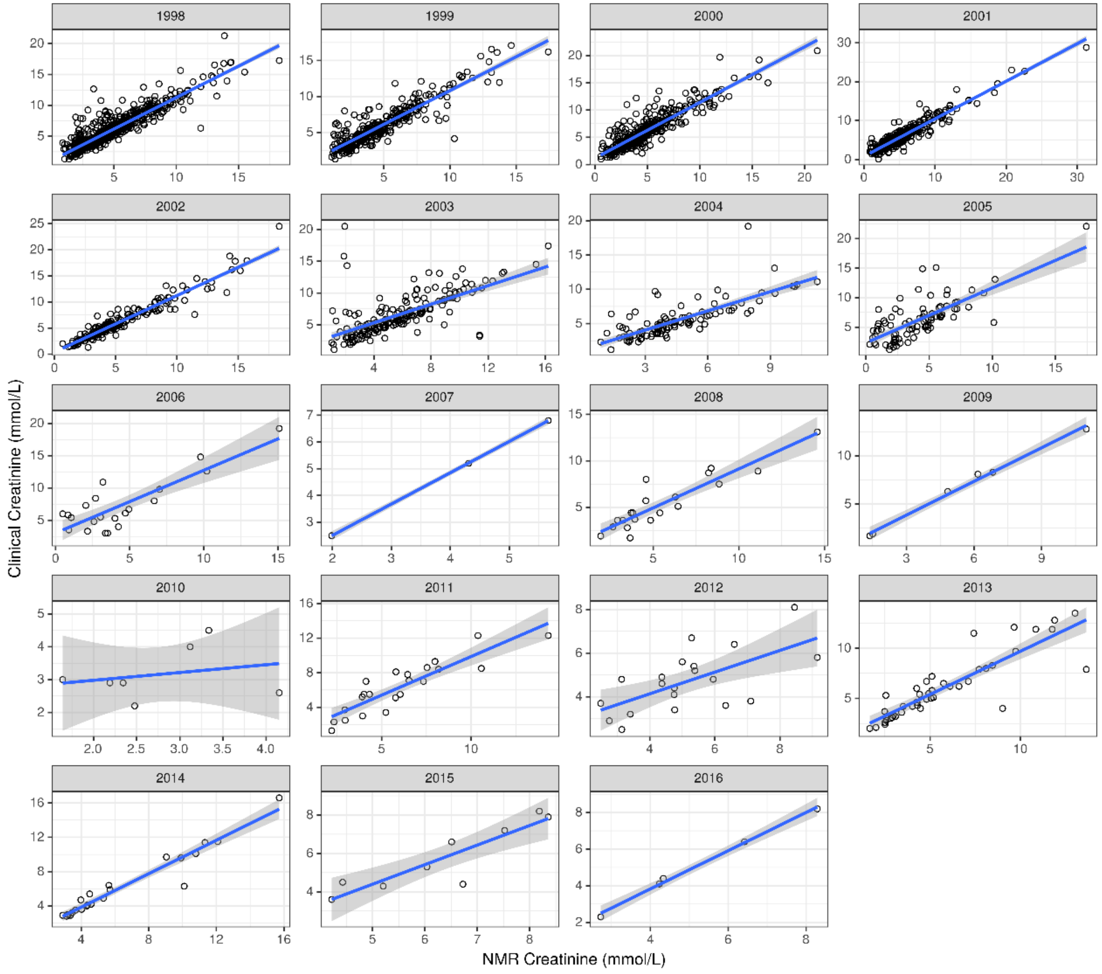

ESM Figure 4. *Correlation of creatinine by clinical chemistry at the time of sampling with creatinine measured by NMR after a median storage time of 17.8 years at −20˚C for 2,628 urine samples from 24h urine collections where data was available grouped by the year of sample taking. The regression lines were fitted with an intercept of 0*

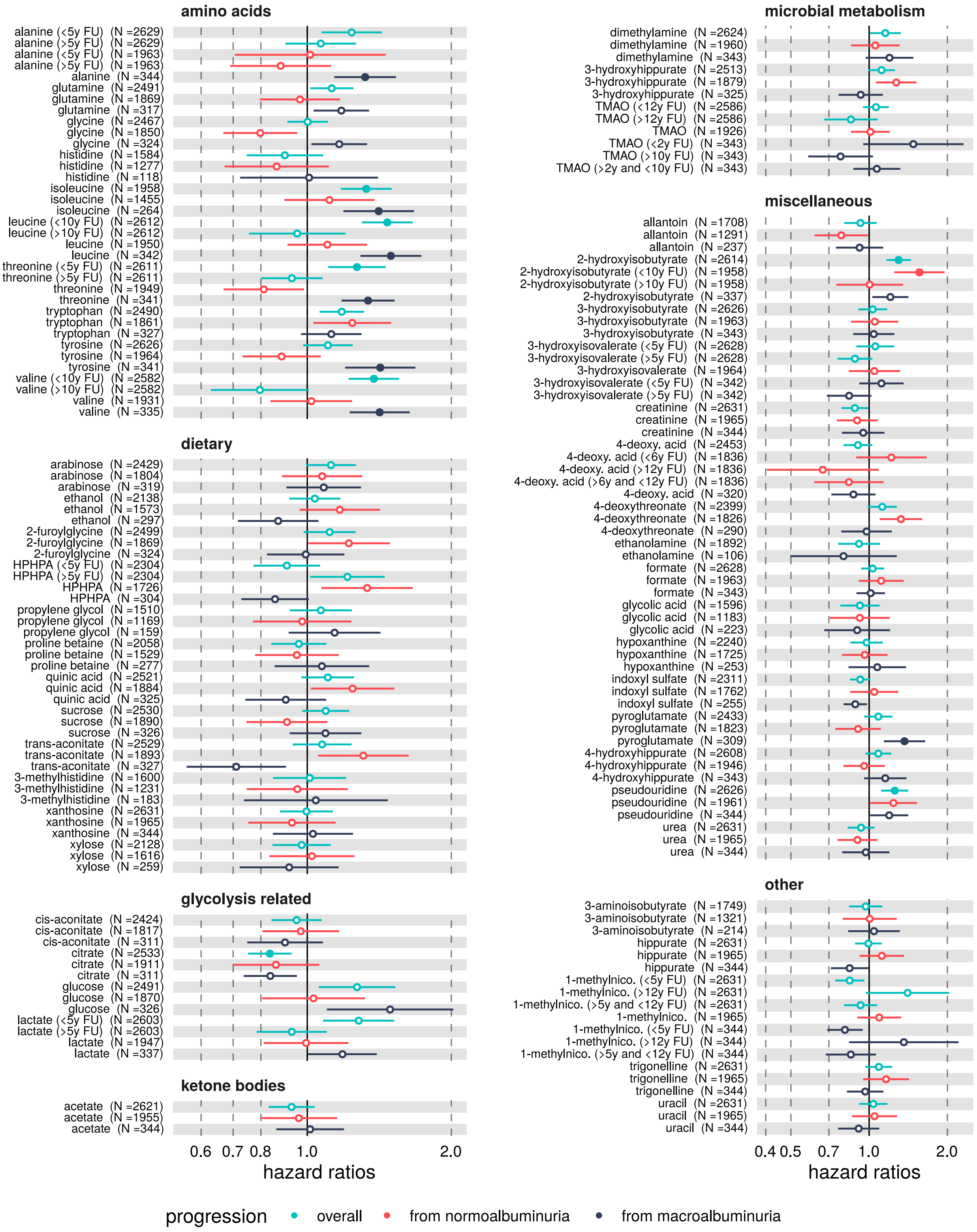

ESM Figure 5. *Standardised hazard ratios per 1-SD increment and 95% confidence intervals for urinary metabolites associated with progression of diabetic nephropathy including overall progression and*

progression from normo- and macroalbuminuria. Urine metabolites were scaled to creatinine and log-transformed. The analysis was adjusted for sex and baseline age, year of diabetes diagnosis, baseline glycemic control (HbA_{1c} > 58.5 mmol/mol or 7.5%) and baseline CKD stage and albuminuria class (in overall progression only). Hazard ratios were scaled to SD-units. A p-value below 0.001 denotes statistical significance and is displayed by a full circle. The proportional hazard assumption was tested with Schoenfeld residuals and follow-up times were split when violated. Other: nicotinate, nicotinamide-, pyrimidine- or phenylalanine metabolism; FU: follow-up; y: years; 4-deoxy. acid: 4-dexoyerythronic acid; 1-methylnico.: 1-methylnicotinamide; HPHPA: 3-(3-hydroxyphenyl)-3-hydroxypropionic acid; TMAO: trimethylamine-N-oxide.

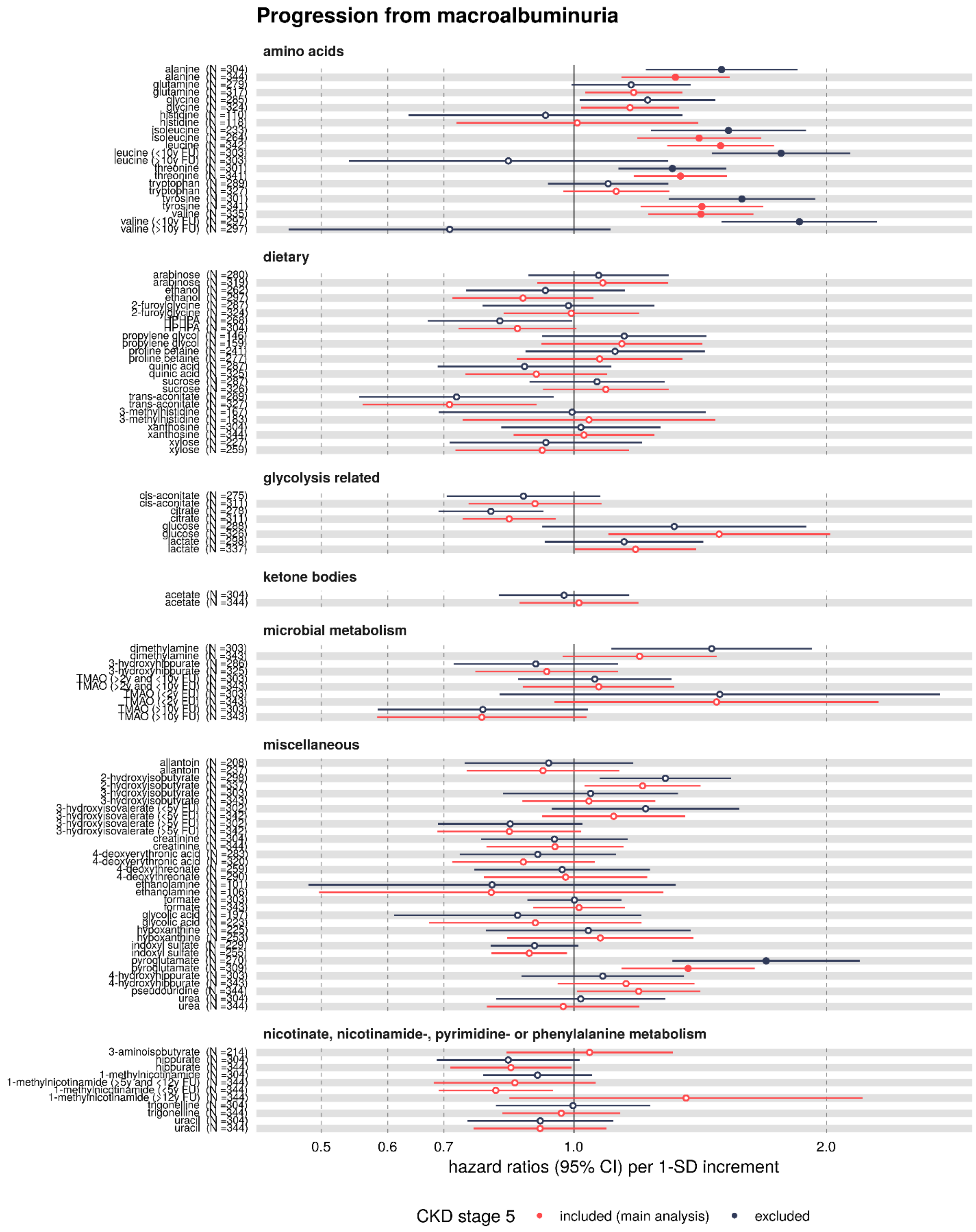

ESM Figure 6. *Standardised hazard ratios and 95% confidence intervals for urinary metabolites associated with progression from macroalbuminuria by firstly including all individuals with macroalbuminuria and secondly excluding those individuals with macroalbuminuria in CKD stage 5. Urine metabolites were scaled*

to creatinine and log-transformed. The analysis was adjusted for sex and baseline age, year of diabetes diagnosis, baseline glycemic control (HbA_{1c} > 58.5 mmol/mol or 7.5%) and baseline CKD stage. Hazard ratios were scaled to SD-units. A p-value below 0.001 denotes statistical significance and is displayed by a full circle. The proportional hazard assumption was tested with Schoenfeld residuals and follow-up times were split when violated. FU: follow-up; y: years; HPHPA: 3-(3-hydroxyphenyl)-3-hydroxypropionic acid; TMAO: trimethylamine-N-oxide.

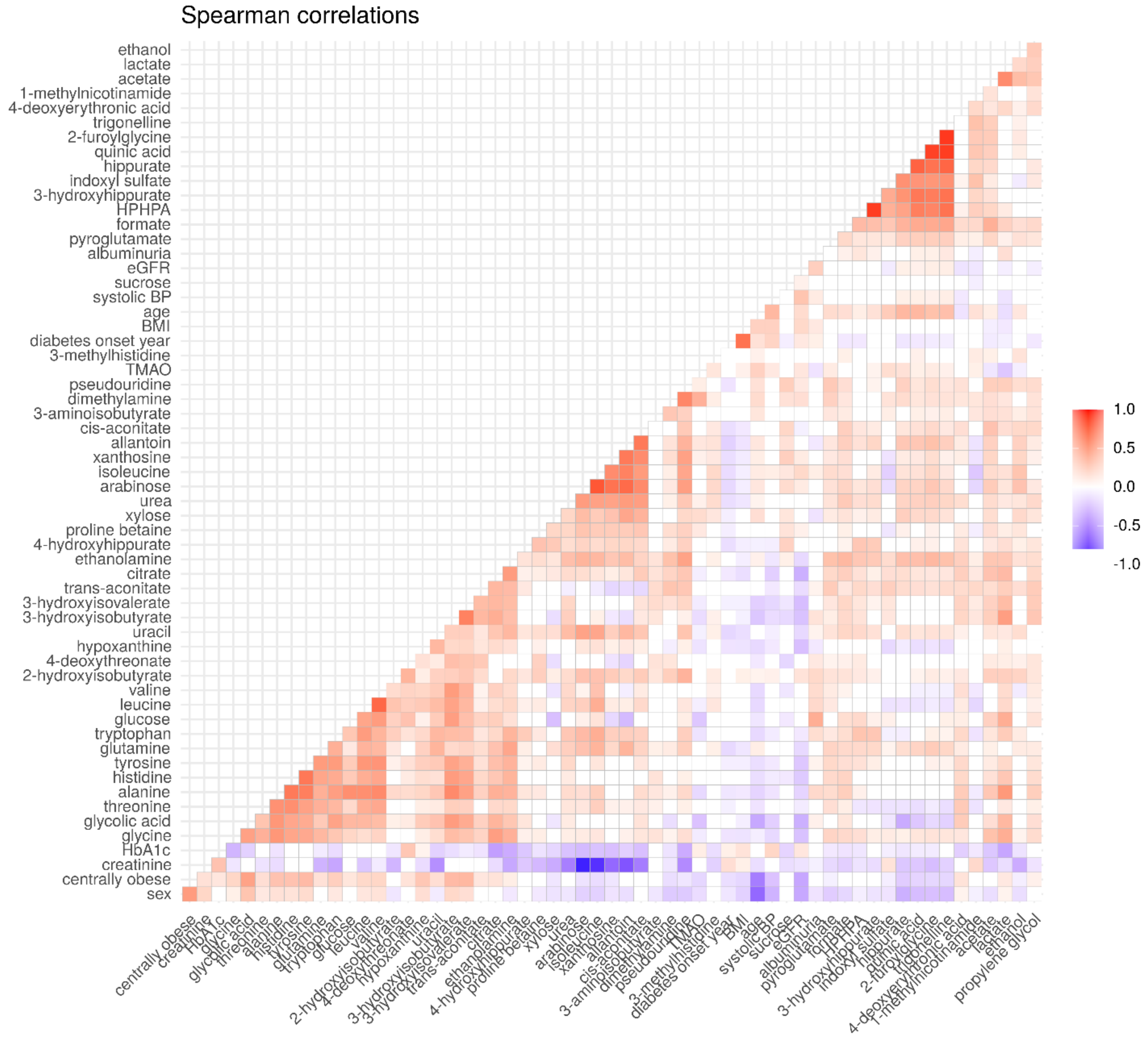

ESM Figure 7. *Spearman correlations of urinary metabolites and clinical covariates used in the Cox Regressions. The variables were ordered based on hierarchical clustering.*

ESM 5: FINNDIANE STUDY CENTERS

The Finnish Diabetic Nephropathy Study Center	Physicians and nurses
Anjalankoski Health Center	S.Koivula, T.Uggeldahl
Central Finland Central Hospital, Jyväskylä	T.Forslund, A.Halonen, A.Koistinen, P.Koskiaho, M.Laukkanen, J.Saltevo, M.Tiihonen
Central Hospital of Åland Islands, Mariehamn	M.Forsen, H.Granlund, A.-C.Jonsson, B.Nyroos
Central Hospital of Kanta-Häme, Hämeenlinna	P.Kinnunen, A.Orvola, T.Salonen, A.Vähänen
Central Hospital of Kymenlaakso, Kotka	R.Paldanius, M.Riihelä, L.Ryysy
Central Hospital of Länsi-Pohja, Kemi	H.Laukkanen, P.Nyländen, A.Sademies
Central Ostrobothnian Hospital District, Kokkola	S.Anderson, B.Asplund, U.Byskata, P.Liedes, M.Kuusela, T.Virkkala
City of Espoo Health Center:	
Espoonlahti	A.Nikkola, E.Ritola
Tapiola	M.Niska, H.Saarinen
Samaria	E.Oukko-Ruponen, T.Virtanen
Viherlaakso	A.Lyytinen
City of Helsinki Health Center:	
Puistola	H.Kari, T.Simonen
Suutarila	A.Kaprio, J.Kärkkäinen, B.Rantaeskola
Töölö	P.Kääriäinen, J.Haaga, A-L.Pietiläinen
City of Hyvinkää Health Center	S.Klemetti, T.Nyandoto, E.Rontu, S.Satuli-Autere
City of Vantaa Health Center:	
Korso	R.Toivonen, H.Virtanen
Länsimäki	R.Ahonen, M.Ivaska-Suomela, A.Jauhiainen
Martinlaakso	M.Laine, T.Pellonpää, R.Puranen
Myyrmäki	A.Airas, J.Laakso, K.Rautavaara
Rekola	M.Erola, E.Jatkola
Tikkurila	R.Lönnblad, A.Malm, J.Mäkelä, E.Rautamo
Heinola Health Center	P.Hentunen, J.Lagerstam
Helsinki University Central Hospital	M.Feodoroff, D.Gordin, O.Heikkilä, K.Hietala, J.Fagerudd, M.Korolainen, L.Kyllönen, J.Kytö, S.Lindh, K.Pettersson-Fernholm, M.Rosengård-Bärlund, A.Sandelin, L.Thorn, J.Tuomikangas, T.Vesisenaho, J.Wadén
Herttoniemi Hospital, Helsinki	V.Sipilä
Hospital of Lounais-Häme, Forssa	T.Kalliomäki, J.Koskelainen, R.Nikkanen, N.Savolainen, H.Sulonen, E.Valtonen
Hyvinkää Hospital	L. Norvio, A.Hämäläinen
Iisalmi Hospital	E.Toivanen
Jokilaakso Hospital, Jämsä	A.Parta, I.Pirttiniemi
Jorvi Hospital, Helsinki University Central Hospital	S.Aranko, S.Ervasti, R.Kauppinen-Mäkelin, A.Kuusisto, T.Leppälä, K.Nikkilä, L.Pekkonen
Jyväskylä Health Center, Kyllö	K.Nuorva, M.Tiihonen
Kainuu Central Hospital, Kajaani	S.Jokelainen, K.Kananen, M.Karjalainen, P.Kemppainen, A-M.Mankinen, A.Reponen, M.Sankari
Kerava Health Center	H.Stuckey, P.Suominen
Kirkkonummi Health Center	A.Lappalainen, M.Liimatainen, J.Santaholma
Kivelä Hospital, Helsinki	A.Aimolahti, E.Huovinen
Koskela Hospital, Helsinki	V.Ilkka, M.Lehtimäki
Kotka Health Center	E.Pälikkö-Kontinen, A.Vanhanen
Kouvola Health Center	E.Koskinen, T.Siitonen
Kuopio University Hospital	E.Huttunen, R.Ikäheimo, P.Karhapää, P.Kekäläinen, M.Laakso, T.Lakka, E.Lampainen, L.Moilanen, S. Tanskanen, L.Niskanen, U.Tuovinen, I.Vauhkonen, E.Voutilainen
Kuusamo Health Center	T.Kääriäinen, E.Isopoussu
Kuusankoski Hospital	E.Kilkki, I.Koskinen, L.Riihelä
Laakso Hospital, Helsinki	T.Meriläinen, P.Poukka, R.Savolainen, N.Uhlenius
Lahti City Hospital	A.Mäkelä, M.Tanner
Lapland Central Hospital, Rovaniemi	L.Hyvärinen, K.Lampela, S.Pöykkö, T.Rompasaari, S.Severinkangas, T.Tulokas
Lappeenranta Health Center	P. Erola, L.Härkönen, P.Linkola, T.Pekkanen, I.Pulli, E.Repo
Lohja Hospital	T.Granlund, K.Hietanen, M.Porrassalmi, M.Saari, T.Salonen, M.Tiikkainen,
Länsi-Uusimaa Hospital, Tammisaari	I.-M.Jousmaa, J.Rinne
Loimaa Health Center	A.Mäkelä, P.Eloranta
Malmi Hospital, Helsinki	H.Lanki, S.Moilanen, M.Tilly-Kiesi
Mikkeli Central Hospital	A.Gynther, R.Manninen, P.Nironen, M.Salminen, T.Vänttinen
Mänttä Regional Hospital	I.Pirttiniemi, A-M.Hänninen
North Karelian Hospital, Joensuu	U-M.Henttula, P.Kekäläinen, M.Pietarinen, A.Rissanen, M.Voutilainen
Nurmijärvi Health Center	A.Burgos, K.Urtamo

Oulaskangas Hospital, Oulainen	E.Jokelainen, P-L.Jylkkä, E.Kaarlela, J.Vuolaspuro
Oulu Health Center	L.Hiltunen, R.Häkkinen, S.Keinänen-Kiukaanniemi
Oulu University Hospital	R.Ikäheimo
Päijät-Häme Central Hospital	H.Haapamäki, A.Helanterä, S.Hämäläinen, V.Ilvesmäki, H.Miettinen
Palokka Health Center	P.Sopanen, L.Welling
Pieksämäki Hospital	V.Sevtsenko, M.Tamminen
Pietarsaari Hospital	M-L.Holmbäck, B.Isomaa, L.Sarelin
Pori City Hospital	P.Ahonen, P.Merisalo, E.Muurinen, K.Sävelä
Porvoo Hospital	M.Kallio, B.Rask, S.Rämö
Raahe Hospital	A.Holma, M.Honkala, A.Tuomivaara, R.Vainionpää
Rauma Hospital	K.Laine, K.Saarinen, T.Salminen
Riihimäki Hospital	P.Aalto, E.Immonen, L.Juurinen
Salo Hospital	A.Alanko, J.Lapinleimu, P.Rautio, M.Virtanen
Satakunta Central Hospital, Pori	M.Asola, M.Juhola, P.Kunelius, M.-L.Lahdenmäki, P.Pääkkönen, M.Rautavirta
Savonlinna Central Hospital	T.Pulli, P.Sallinen, M.Taskinen, E.Tolvanen, T.Tuominen, H.Valtonen, A.Vartia, S-L. Viitanen
Seinäjoki Central Hospital	O.Antila, E.Korpi-Hyövälti, T.Latvala, E.Leijala, T.Leikkari, M.Punkari, N.Rantamäki, H.Vähävuori
South Karelia Central Hospital, Lappeenranta	T.Ensala, E.Hussi, R.Härkönen, U.Nyholm, J.Toivanen
Tampere Health Center	A.Vaden, P.Alarotu, E.Kujansuu, H.Kirkkopelto-Jokinen, M.Helin, S.Gummerus, L.Calonius, T.Niskanen, T.Kaitala, T.Vatanen
Tampere University Hospital	P. Hannula, I.Ala-Houhala, R.Kannisto, T.Kuningas, P.Lampinen, M.Määttä, H.Oksala, T.Oksanen, A.Putila, H.Saha, K.Salonen, H.Tauriainen, S.Tulokas
Tiirismaa Health Center, Hollola	T.Kivelä, L.Petlin, L.Savolainen
Turku Health Center	A.Artukka, I.Hämäläinen, L.Lehtinen, E.Pyysalo, H.Virtamo, M.Viinikkala, M.Vähätalo
Turku University Central Hospital	K.Breitholz, R.Eskola, K.Metsärinne, U.Pietilä, P.Saarinen, R.Tuominen, S.Äyräpää
Vaajakoski Health Center	K.Mäkinen, P.Sopanen
Valkeakoski Regional Hospital	S.Ojanen, E.Valtonen, H.Ylönen, M.Rautiainen,T.Immonen
Vammala Regional Hospital	I.Isomäki, R.Kroneld, L.Mustaniemi, M.Tapiolinna-Mäkelä
Vaasa Central Hospital	S.Bergkulla, U.Hautamäki, V-A.Myllyniemi, I.Rusk

LIST OF ABBREVIATIONS IN ESM FIGURE 1

Here is a list of abbreviation for metabolite names used in ESM Figure 1:

ace	Acetate
ala	Alanine
aln	Allantoin
aohibut	2-Hydroxyisobutyrate
arb	Arabinose
bohibut	3-Hydroxyisobutyrate
bohival	3-Hydroxyisovalerate
caco	cis-Aconitate
cit	Citrate
crea	Creatinine
dma	Dimethylamine
doeta	4-Deoxyerythronic acid
dta	4-Deoxythreonate
etoh	Ethanol
form	Formate
furgly	2-Furoylglycine
glc	Glucose
gln	Glutamine
gly	Glycine
glya	Glycolic acid
hip	Hippurate
hphpa	3-(3-Hydroxyphenyl)-3-hydroxypropionic acid
hyp	Hypoxanthine
ile	Isoleucine
ind	Indoxyl Sulfate
lac	Lactate
leu	Leucine
mohhip	3-hydroxyhippurate
omna	1-Methylnicotinamide
pglu	Pyroglutamate
pohhip	4-Hydroxyhippurate
probet	Proline betaine
pseur	Pseudouridine
quina	Quinic acid
scr	Sucrose
taco	trans-Aconitate
thre	Threonine
tmao	Trimethylamine-N-oxide
tmehis	3-Methylhistidine
trig	Trigonelline
trp	Tryptophan
tyr	Tyrosine

ura	Uracil
ure	Urea
val	Valine
xan	Xanthosine
xyl	Xylose

ESM REFERENCES

1. Elliott P, Posma JM, Chan Q, et al (2015) Urinary metabolic signatures of human adiposity. Sci Transl Med 7(285):285ra62. https://doi.org/10.1126/scitranslmed.aaa5680

2. Bouatra S, Aziat F, Mandal R, et al (2013) The Human Urine Metabolome. PLOS ONE 8(9):e73076. https://doi.org/10.1371/journal.pone.0073076

3. Weljie AM, Newton J, Mercier P, Carlson E, Slupsky CM (2006) Targeted Profiling: Quantitative Analysis of 1H NMR Metabolomics Data. Anal Chem 78(13):4430–4442. https://doi.org/10.1021/ac060209g

4. Holmes E, Foxall PJD, Spraul M, Duncan Farrant R, Nicholson JK, Lindon JC (1997) 750 MHz 1H NMR spectroscopy characterisation of the complex metabolic pattern of urine from patients with inborn errors of metabolism: 2-hydroxyglutaric aciduria and maple syrup urine disease. J Pharm Biomed Anal 15(11):1647–1659. https://doi.org/10.1016/S0731-7085(97)00066-6

5. Soininen P, Kangas AJ, Würtz P, Suna T, Ala-Korpela M (2015) Quantitative Serum Nuclear Magnetic Resonance Metabolomics in Cardiovascular Epidemiology and Genetics. Circ Cardiovasc Genet 8(1):192–206. https://doi.org/10.1161/CIRCGENETICS.114.000216

6. Tynkkynen T, Wang Q, Ekholm J, et al (2019) Proof of concept for quantitative urine NMR metabolomics pipeline for large-scale epidemiology and genetics. Int J Epidemiol 48(3):978–993. https://doi.org/10.1093/ije/dyy287

7. Xiao C, Hao F, Qin X, Wang Y, Tang H (2009) An optimized buffer system for NMR-based urinary metabonomics with effective pH control, chemical shift consistency and dilution minimization. Analyst 134(5):916–925. https://doi.org/10.1039/B818802E

8. Soininen P, Haarala J, Vepsäläinen J, Niemitz M, Laatikainen R (2005) Strategies for organic impurity quantification by 1H NMR spectroscopy: Constrained total-line-shape fitting. Anal Chim Acta 542(2):178–185. https://doi.org/10.1016/j.aca.2005.03.060

9. Emwas A-H, Roy R, McKay RT, et al (2016) Recommendations and Standardization of Biomarker Quantification Using NMR-Based Metabolomics with Particular Focus on Urinary Analysis. J Proteome Res 15(2):360–373. https://doi.org/10.1021/acs.jproteome.5b00885
